# Novel and Annotated Long Noncoding RNAs Associated with Ischemia in the Human Heart

**DOI:** 10.3390/ijms222111324

**Published:** 2021-10-20

**Authors:** Zoe Ward, Sebastian Schmeier, Louis Saddic, Martin I. Sigurdsson, Vicky A. Cameron, John Pearson, Allison Miller, Arthur Morley-Bunker, Josh Gorham, Jonathan G. Seidman, Christine S. Moravec, Wendy E. Sweet, Sary F. Aranki, Simon Body, Jochen D. Muehlschlegel, Anna P. Pilbrow

**Affiliations:** 1 Christchurch Heart Institute, University of Otago, Christchurch 8011, New Zealand; vicky.cameron@otago.ac.nz (V.A.C.); anna.pilbrow@otago.ac.nz (A.P.P.); 2 School of Natural and Computational Sciences, Massey University, Auckland 0745, New Zealand; s.schmeier@gmail.com; 3 Department of Anesthesiology and Perioperative Medicine, David Geffen School of Medicine, University of California, Los Angeles, CA 90095, USA; lousaddic@gmail.com; 4 Department of Anesthesiology and Critical Care Medicine, Landspitali—The National University Hospital of Iceland, Faculty of Medicine, University of Iceland, 101 Reykjavik, Iceland; mingi@hi.is; 5 Biostatistics and Computational Biology Unit, University of Otago, Christchurch 8011, New Zealand; john.pearson@otago.ac.nz; 6 Department of Pathology and Biomedical Science, University of Otago, Christchurch 8011, New Zealand; allison.miller@otago.ac.nz (A.M.); arthur.morley-bunker@otago.ac.nz (A.M.-B.); 7 Department of Genetics, Harvard Medical School, Boston, MA 02115, USA; jgorham@genetics.med.harvard.edu (J.G.); seidman@genetics.med.harvard.edu (J.G.S.); 8 Heart and Vascular Institute, Cleveland Clinic, Cleveland, OH 44122, USA; moravec@ccf.org (C.S.M.); sweetw@ccf.org (W.E.S.); 9 Department of Surgery, Division of Cardiac Surgery, Brigham and Women’s Hospital, Harvard Medical School, Boston, MA 02115, USA; saranki@bwh.harvard.edu (S.F.A.); JMUEHLSCHLEGEL@BWH.HARVARD.EDU (J.D.M.); 10 Department of Anesthesiology, Boston University School of Medicine, Boston, MA 02115, USA; scbody@bu.edu

**Keywords:** myocardial ischemia, long noncoding RNA, RNA sequencing

## Abstract

Background: Long noncoding RNAs (lncRNAs) have been implicated in the pathogenesis of cardiovascular diseases. We aimed to identify novel lncRNAs associated with the early response to ischemia in the heart. Methods and Results: RNA sequencing data gathered from 81 paired left ventricle samples from patients undergoing cardiopulmonary bypass was collected before and after a period of ischemia. Novel lncRNAs were validated with Oxford Nanopore Technologies long-read sequencing. Gene modules associated with an early ischemic response were identified and the subcellular location of selected lncRNAs was determined with RNAscope. A total of 2446 mRNAs, 270 annotated lncRNAs and one novel lncRNA differed in response to ischemia (adjusted *p* < 0.001, absolute fold change >1.2). The novel lncRNA belonged to a gene module of highly correlated genes that also included 39 annotated lncRNAs. This module associated with ischemia (Pearson correlation coefficient = −0.69, *p* = 1 × 10^−23^) and activation of cell death pathways (*p* < 6 × 10^−9^). A further nine novel cardiac lncRNAs were identified, of which, one overlapped five cis-eQTL eSNPs for the gene RWD Domain-Containing Sumoylation Enhancer (RWDD3) and was itself correlated with RWDD3 expression (Pearson correlation coefficient −0.2, *p* = 0.002). Conclusion: We have identified 10 novel lncRNAs, one of which was associated with myocardial ischemia and may have potential as a novel therapeutic target or early marker for myocardial dysfunction.

## 1. Introduction

Approximately 80% of the human genome is transcribed, with only approximately 3% belonging to protein-coding transcripts [1]. Transcripts that lack protein-coding potential have been classified into either small noncoding RNAs, such as microRNAs (miRNAs), small interfering RNAs (siRNAs), and Piwi-interacting RNAs (piRNAs), or long noncoding RNAs (lncRNAs) for transcripts >200 nucleotides. LncRNAs share some similarities with mRNAs—they are both transcribed by RNA polymerase II (RNAPII) from loci with similar epigenetic marks (H3K4me3) at their promoter regions and are mostly thought to be 5′7-methylguanosine capped, spliced and have and a 3′poly(A) tail [2]. However, lncRNAs generally have lower expression levels (10-fold lower) and appear to show a high degree of specificity for cell and tissue type, developmental stage and disease state [3]. LncRNAs have less conserved primary sequences, although this level of conservation is higher at lncRNA exon splice sites and much higher at their promoters. To date, depending on the choice of annotation, there are between 17,910 (GENCODE v32), 27,919 (FANTOM5 [4]) and 96,308 (NONCODE v5.0) human lncRNA genes. Functional characterization of this ever-expanding class is lagging behind their discovery and annotation. Despite this, many lncRNAs have been implicated in a wide range of biological processes and in various diseases including cardiovascular disease [5].

Accumulation of atherosclerotic plaque in the coronary arteries can restrict blood supply to the heart, leading to myocardial ischemia and myocardial infarction. While cardiac troponins have emerged as vital biomarkers in the clinical diagnosis of myocardial infarction, there is a lack of specific early markers for myocardial ischemia that could help identify cell dysfunction before damage has become irreversible [6]. Such biomarkers would add to current clinical tools and may help improve assessment of pre-morbid myocardial dysfunction in asymptomatic individuals, facilitating better monitoring and early use of preventative strategies.

We hypothesize that long noncoding RNAs help coordinate the post-ischemic stress response and that advanced bioinformatics tools may identify novel lncRNAs not previously annotated. Previously, [7] RNA sequencing (RNA-seq) performed after a period of ischemia in left ventricular human heart tissue before and after cardiopulmonary bypass identified several lncRNAs differentially expressed annotated lncRNAs in response to ischemia in the human heart. However, unannotated lncRNAs involved in the response to ischemia were not described. Here, we describe a bioinformatics pipeline to interrogate RNA-seq data from human hearts pre- and post-ischemia to identify potential novel lncRNA biomarkers associated with cardiac ischemia.

## 2. Results

### 2.1. Protein Coding, Annotated and Novel lncRNAs Associated with Ischemia Identified with Illumina Short-Read Sequencing

**Figure 1:**
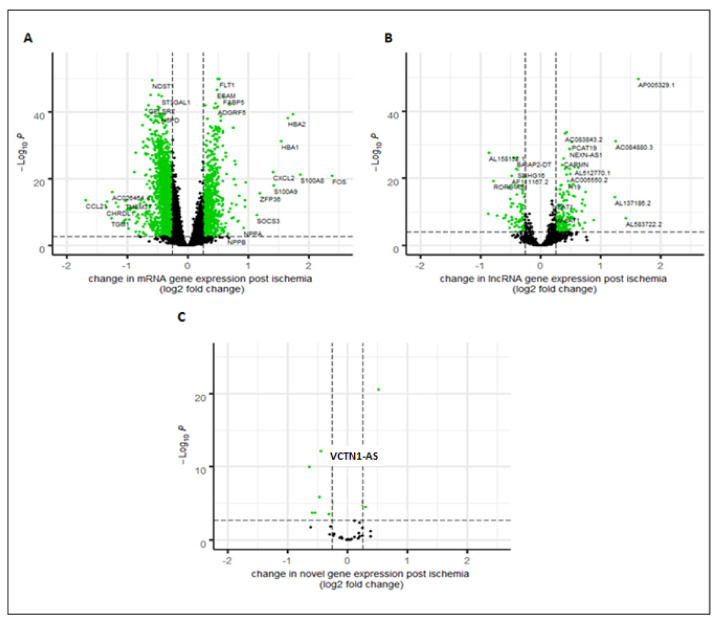
Volcano plots showing differential expression in 81 paired human left ventricle samples, comparing pre- versus post-ischemia: (**A**). mRNA genes, (**B**). annotated long noncoding RNA (lncRNA) genes, and (**C**). putative novel lncRNA transcripts. Green indicates differentially expressed genes/transcripts adjusted *p* value (padj) < 0.001 with an absolute fold change >1.2.

Illumina RNA-seq generated an average of 33.7 ± 12.6 million uniquely mapped reads per sample (approximately 85% of the total reads per sample). Unique reads were mapped to 12,656 protein-coding genes (mRNAs), 1488 annotated lncRNA genes (Figure 1A,B) and 10,567 putative novel lncRNAs in the human left ventricle. Among the annotated lncRNA genes were known cardiac-related lncRNAs, MALAT1, NEAT1, H19, and TUG1 [8,9,10,11]. Expression levels of 2446 mRNAs, 270 annotated lncRNAs and 1137 putative novel lncRNAs met the prespecified criteria for an association with ischemia.

### 2.2. Validating Novel lncRNAs in the Human Left Ventricle with Nanopore Long-Read Technology

Nanopore long-read RNA sequencing on an independent pooled sample of 8 human left ventricle tissues yielded a total of 10,638,219 base-called reads (Supplementary Figure S5). Of these, 2,710,486 full-length transcripts were identified, which had an alignment rate of 96.3% (75.6% to hg38 and 20.7% to chrIS). From these transcripts, 153 potential multi-exonic, putative novel lncRNAs were identified.

**Figure 2:**
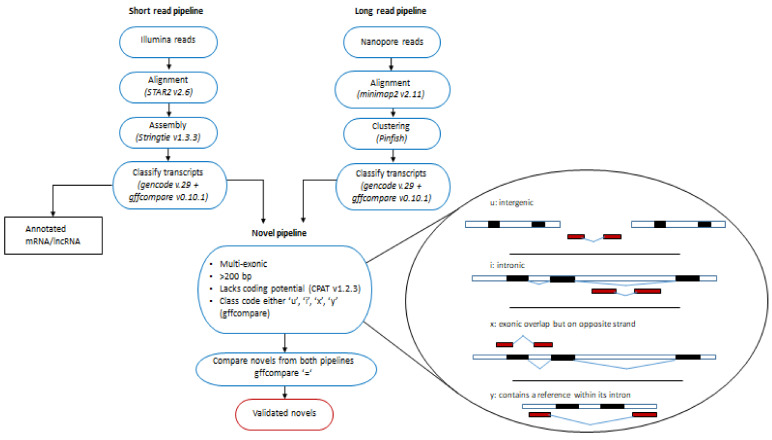
A schematic of the pipeline for novel lncRNA discovery (Illumina sequencing) and validation (Nanopore sequencing).

Comparison of the novel lncRNA transcripts identified by Illumina RNA-seq and Nanopore RNA-seq identified 38 transcripts from 34 unique genes in common with both sequencing platforms (four transcripts represented different isoforms) that shared complete intron chains (gffcompare class code “=”, Figure 2). Of these, 19 were intergenic (gffcompare class code ‘u’), 13 were intronic (‘i’), five were novel antisense (‘x’) and one contained a reference gene within its intron (‘y’, class codes, Figure 2).

As our pipeline was established using GENCODE v.29, confirmation of the 38 putative novel lncRNAs was determined by searching the updated version of GENCODE (v.32), and lncRNA databases, FANTOM CAT http://fantom.gsc.riken.jp/cat/?fd=source_data (accessed on September 2019) and NONCODE v.5. Of these, 28 had been previously reported: 12 transcripts (from 10 unique lncRNA genes) in GENCODE v.32, 21 transcripts (from 19 unique lncRNA genes) in FANTOM CAT and 15 transcripts (from 12 unique lncRNA genes) in NONCODE v.5. The 28 replicated lncRNAs confirmed the validity of our discovery pipeline and identified 10 remaining novel lncRNAs not previously described (Figure 1C). We have putatively named these novel lncRNAs according to the guidelines according to Seal et al. 2020 [12]. Table 1 lists these novel lncRNAs with their names, chromosome positions and the mean TPMs (for chromosome positions of each exon see Supplementary Table S1). Of these, one was differentially expressed between pre- and post-ischemia (fold change −1.4, adjusted *p*-value = 7.27 × 10^−13^). When viewed in the Ensembl Genome Browser this transcript overlaps an enhancer, a transcription factor-binding site and two CTCF sites (Supplementary Figure S4A).

### 2.3. Evolutionary Conservation of lncRNAs

**Figure 3:**
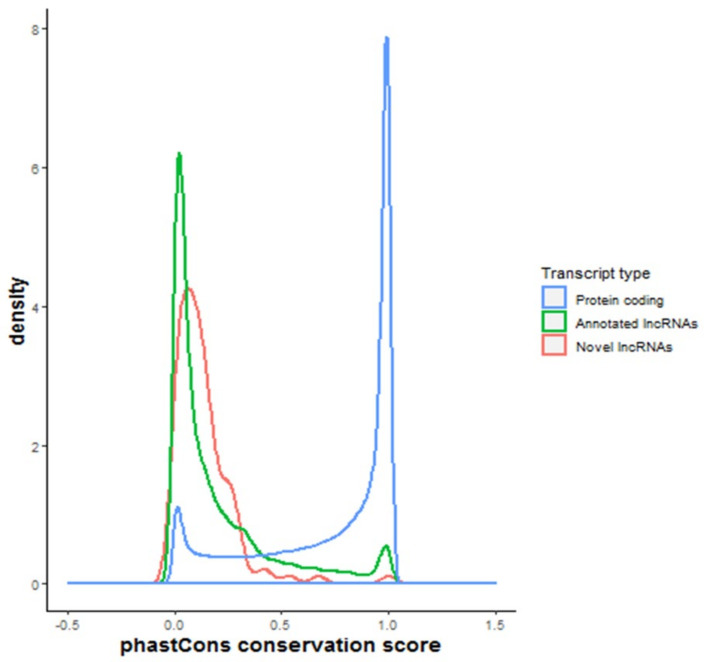
Geometric density plots showing the frequency of phastCons conservation scores for 20 mammalian species averaged base-wise for each exon. Exons from protein-coding transcripts (expressed in this study) are shown in blue, the exons from annotated lncRNAs (expressed in this study) are shown in green and 10 identified novel lncRNAs are shown in red. The phastCons score estimates the probability that a nucleotide is conserved; the closer the score is to 1, the more conserved the base.

To test whether our novel lncRNAs showed similar profiles of conservation to annotated lncRNAs, the averaged, pre-computed, per-base evolutionary conservation scores for each exon were compared. The novel lncRNAs had low primary sequence conservation similar to annotated lncRNAs and a markedly different profile from protein-coding genes (Figure 3).

### 2.4. Identifying Gene Networks Associated with Ischemia

WGCNA identified 18 modules of highly correlated genes with similar expression profiles across patients. Of these, two were strongly associated with ischemia (Pearson correlation coefficient (PCC) 0.61, *p*-value < 2 × 10^−17^ − 0.69, *p*-value 1 × 10^−23^, Supplementary Figure S6).

**Figure 4:**
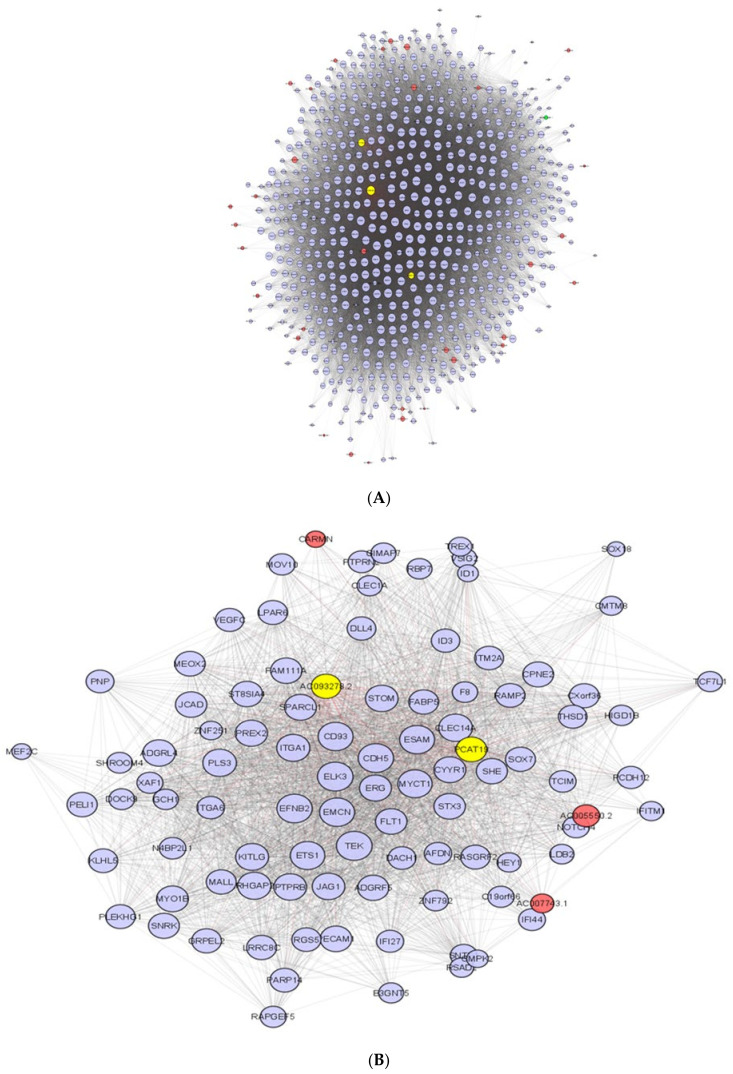
Co-expression network between lncRNAs and mRNAs in each module. (**A**) Module 1 shows genes enriched in pathways associated with cell death, apoptosis, and necrosis. (**B**) Module 2 shows genes predicted to activate pathways involved in angiogenesis and vascular development in the cardiovascular system and proliferation of white blood cells and immune cells. Yellow nodes represent lncRNAs with the highest module membership and putative hub genes; Red nodes represent (annotated) lncRNAs; Green node (Module 1) represents the ischemia associated novel lncRNA; Purple nodes represent mRNAs.

On average, expression of genes in Module 1 decreased from pre- to post-ischemia (PCC = −0.69, *p* = 1 × 10^−23^). Genes in this module (mRNAs = 856, lncRNAs = 67) were predicted to activate pathways associated with apoptosis and necrosis (*p* = 5.6 × 10^−8^ − *p* = 6.5 × 10^−9^, Supplementary Table S2). This module included 66 annotated lncRNAs and one novel lncRNA (VTCN1-AS). Three lncRNAs (AC005523.2, AF111167.2 and CTBP1-DT) had module membership correlation values >0.7, suggesting they may act as network hubs. When viewed in the Ensembl genome browser they all overlap or are very close to regulatory elements. AC005523.2 is 145 bases from a CTCF-binding site, AF111167.2 is 683 bases from an enhancer and 2860 from a CTCF-binding site and CTBP1-DT overlaps an enhancer and 6 CTCF-binding sites (Supplementary Figure S4B,D). Module 1 is presented in Figure 4A with the putative hub lncRNAs highlighted.

Expression of genes in Module 2 increased from pre- to post-ischemia (Pearson Correlation coefficient module eigengene with ischemia = 0.67, *p* = 2 × 10^−17^). Genes in Module 2 (mRNAs = 148, lncRNAs = 8) were predicted to activate pathways involved in angiogenesis and vascular development in the cardiovascular system and promote proliferation of white blood cells and immune cells in response to ischemia (*p* = 1.4 × 10^−6^, *p* = 1.0 × 10^−13^, Supplementary Table S2). Of the 8 lncRNAs in Module 2, two (PCAT19 and AC093278.2) had module membership correlation values > 0.7, suggesting they may serve as network hubs. PCAT19 overlaps an enhancer and 6 CTCF-binding sites, AC093278.2 is 1103 bases away from an enhancer (Supplementary Figure S4E,F) There were no novel lncRNAs present in Module 2. Module 2 is presented in Figure 4B with the putative hub lncRNAs highlighted.

### 2.5. Overlap of Annotated and Novel lncRNAs with Cis-eQTLs

To identify possible functional mechanisms of lncRNAs associated with ischemia, we investigated whether any of the annotated or novel lncRNAs overlapped cis-eQTLs in left ventricle tissue in the GTEx database (https://www.gtexportal.org/home/ SNP-gene associations *p*-value < 1 × 10^−8^ accessed on September 2019). In Module 1, 12 annotated lncRNAs overlapped cis-eQTL SNPs. The differentially expressed lncRNA cis-eQTL eGene associations were: AC005523.2- Fem-1 homolog A (FEM1A), AC011476.3—retinol dehydrogenase 13 (RDH13) and AC012313.1—Zinc finger protein 84 (ZNF584). Expression of AC005523.2- FEM1A and AC011476.3—RDH13 was highly correlated (PCC = 0.87 and 0.8; *p* = 1.52 × 10^−51^ and *p* = 1.83 × 10^−37^, respectively (Supplementary Figure S7). However, the correlation of AC012313.1—ZNF584 was not significant. None of the annotated lncRNAs in Module 2 overlapped any eQTL eSNPs.

Of the 10 novel lncRNAs, the two exons of MSTRG.8333.38 overlapped five cis-eQTL eSNPs (rs259352, rs259354, rs3820345, rs10783001 and rs35070110) associated with expression of the gene RWD Domain-Containing Sumoylation Enhancer (RWDD3) (GTEx SNP-gene associations *p* < 1 × 10^−7^). This lncRNA is situated immediately downstream of RWDD3 and was weakly correlated with expression of RWDD3 (PCC −0.2, *p* = −0.002 Supplementary Figure S8) indicating a potential mechanistic link between eSNPs and RWDD3.

### 2.6. Subcellular Localization of Ischemia-Associated lncRNAs with RNA Scope

**Figure 5:**
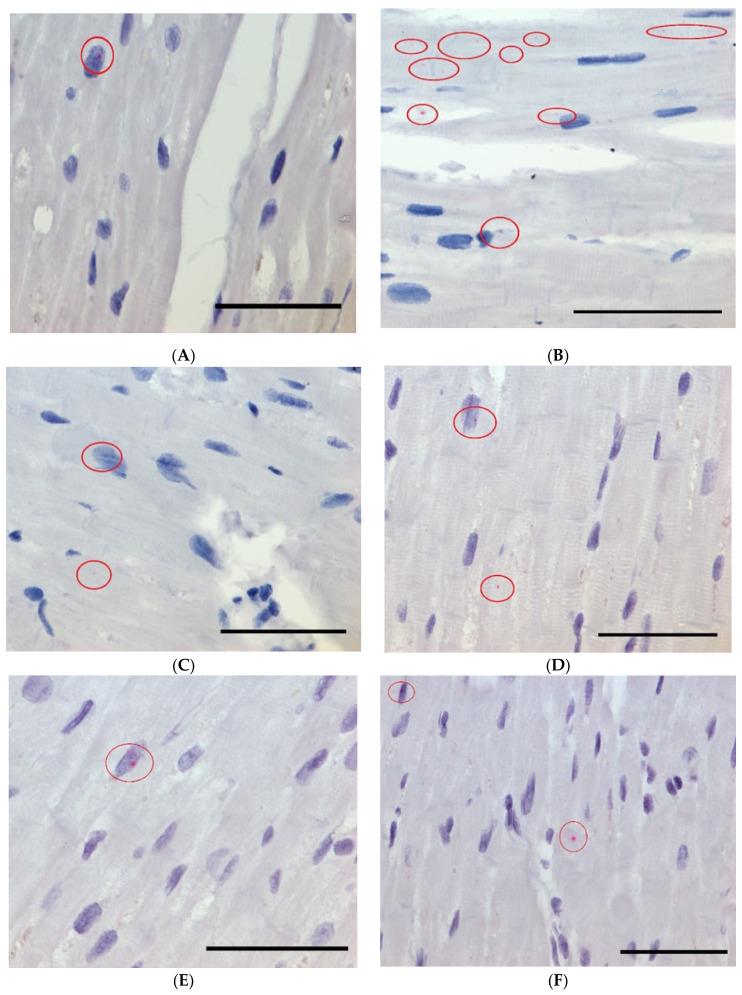

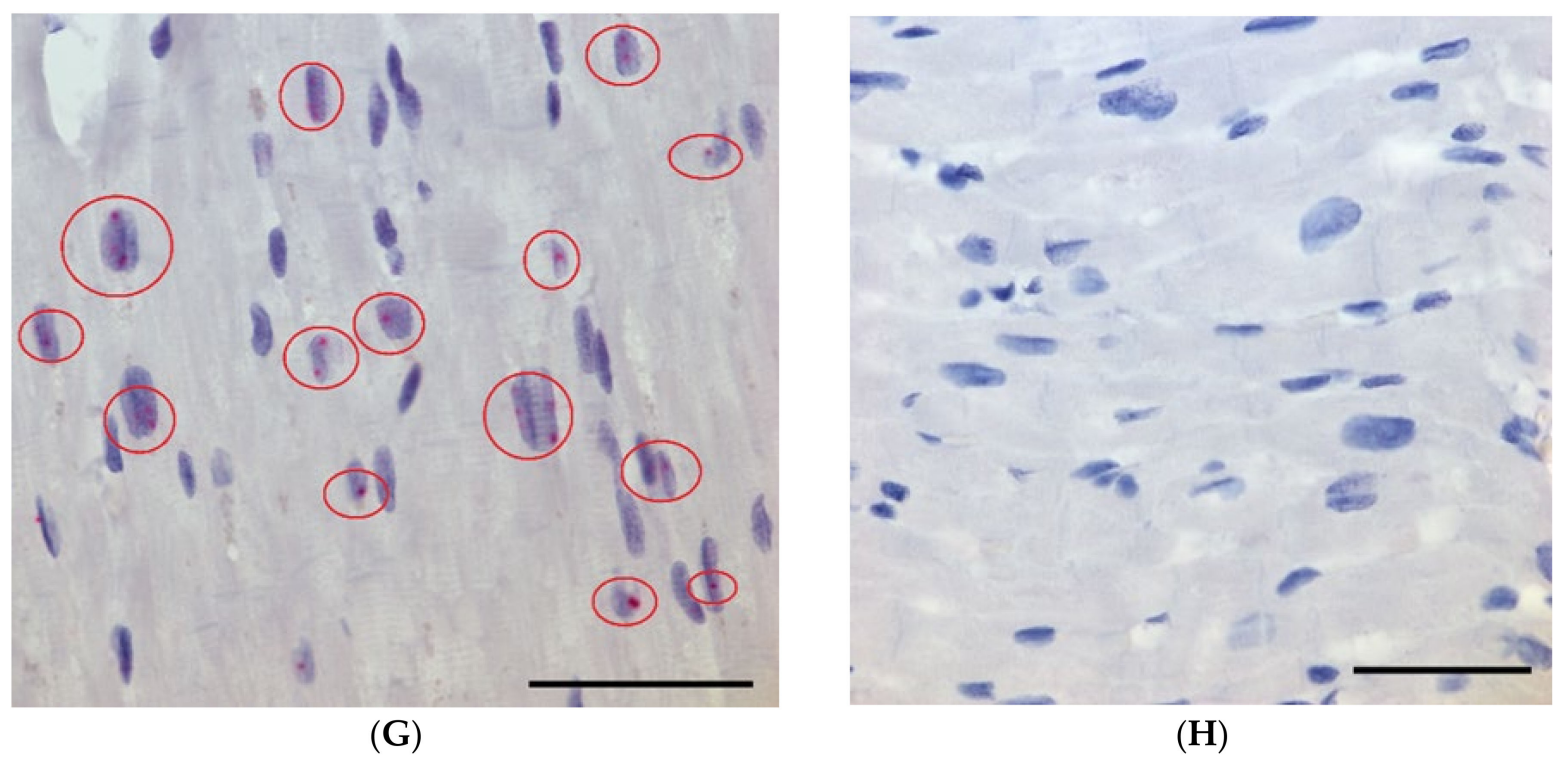
RNA Scope showing VASH1-AS1, PCAT19 and the novel VTCN1-AS expression in cardiomyocytes. The dark blue haematoxylin staining indicates the cell nucleus. Red circles indicate the location of the red RNA-Scope probes. (**A**) VASH1-AS1 probe nuclear localization at 63× magnification, (**B**) VASH1-AS1 probes cytoplasmic localization at 63× magnification (**C**) and (**D**) Shows PCAT19 probe nuclear and cytoplasmic localization at 63× magnification (**E**) and (**F**) Shows novel (VTCN1-AS) probe nuclear and cytoplasmic localization at 63× magnification (**G**) NEAT1 lncRNA positive control with an exclusively nuclear location (**H**) Negative control Scale bar = 50 µm.

RNAscope analysis revealed that two annotated lncRNAs:VASH1-AS1; Module 1, and PCAT19; Module 2, and one novel lncRNA: VTCN1-AS; Module 1 localize both within the nucleus and in the cytoplasm of nearby surrounding tissue (Figure 5).

## 3. Discussion

We have identified ten novel lncRNAs in human left ventricular tissue using a strategy of both short- and long-read sequencing. We have also provided transcript and exon co-ordinates in order for other researchers to validate these novel lncRNAs in their own studies. Of these, the expression of the novel lncRNA VTCN1-AS, along with 270 previously annotated lncRNAs, was altered in response to mild ischemia. Analysis of co-expression networks of lncRNAs and mRNAs identified two modules of genes that may promote angiogenesis, neovascularisation and cardiomyocyte cell death and form part of the early response to myocardial ischemia. Our data suggest lncRNAs are among the first genes to respond to ischemic changes in the left ventricle and may be acting as hub genes to regulate many other mRNAs in the same co-expression network. They may represent novel therapeutic targets or candidate biomarkers for early myocardial dysfunction. This study extends our earlier findings [7] by detecting novel lncRNAs and using a gene network approach to investigate associations between lncRNAs and mRNAs associated with ischemia.

Because lncRNA transcripts are expressed at lower levels compared with mRNAs, many novel lncRNAs may be difficult to detect using short-read sequencing approaches which require use of strict abundance filters to remove noise and minimise the high rate of false positive detection. Here, we have developed a lncRNA identification strategy that involved validating an unfiltered list of putative lncRNAs (identified by short-read sequencing) with long-read sequencing and selection of only multi-exonic transcripts. It was encouraging to see that out of 39 transcripts initially identified as novel, 28 had been documented as lncRNAs in a more recent version of the GENCODE annotation, confirming the validity of our pipeline. Using this approach, we identified 10 novel multi-exonic lncRNAs that were robustly detectable in left ventricle samples, including one novel lncRNA that was altered in response to ischemia. The conservation profile of these novel lncRNAs was similar to known lncRNAs and differed from protein-coding transcripts, suggesting these are genuine noncoding RNAs. The number of genuinely novel transcripts detected by Illumina sequencing but not detected by Nanopore sequencing (and vice versa) remains unknown, and it is likely additional novel transcripts will continue to be discovered in human heart as sequencing technologies improve. Presently, most novel detection pipelines, including ours, filter out single-exon transcripts to minimise false positives. Biologically, there is no reason to do this as some lncRNAs, such as MALAT1, may include a single exon. This issue may be alleviated with long-read technologies as longer reads are less likely to map to the genome by chance.

It was observed that AC005523.2 lies antisense to FEM1A (also in the same WGCNA module) and overlaps a cis-eQTL SNP associated with FEM1A, suggesting AC005523.2 may act as a cis regulator of FEM1A (Supplementary Figure S6). FEM1A is localized within mitochondria of cardiac muscle, is increased after myocardial infarction in mice [13] and may regulate apoptosis [14]. The second lncRNA hub, AAF111167.2, is antisense to, and overlaps the promoter of, JDP2, a transcription factor associated with maladaptive cardiac remodelling [15], whereas the third lncRNA hub, CTBP1-DT, is antisense to a transcriptional repressor, C-terminal-binding protein 1(CTBP1), which is involved in cell proliferation [16]. Several of the protein-coding genes in Module 1 have already been demonstrated to be associated with cardiac energy metabolism and apoptosis such as 2-oxoglutarate dehydrogenase (OGDH), a potent source of reactive oxygen species (ROS) in cardiac mitochondria [17]. Additionally, in the same module, we identified Mitofusin-2 (MFN2), involved in mitochondrial integrity and protecting cardiomyocytes during ischemia-reperfusion injury [18] and Adenylate Cyclase 9 (ADCY9), involved in cardiac apoptosis [19].

LncRNAs can act in trans as well as cis and a gene correlation network analysis may help identify potential regulatory lncRNAs that influence ischemia by acting on one or many mRNAs through networks (modules). WGCNA clusters genes with highly correlated expression into modules (summarized by the module eigengene) and identifies the genes with the highest intra-module connectivity (hub genes) that may regulate the expression of other genes in the module. Using this tool, we have identified three hub lncRNAs (AC005523.2, AF111167.2, CTBP1-DT) within a large gene module that was strongly associated with ischemia and may promote activation of apoptosis and necrosis pathways. While the mechanisms by which these lncRNAs might influence gene expression are unknown, all of the putative hub lncRNAs overlap or are next to regulatory elements of the genome that would suggest a trans mechanism for these lncRNAs regulating other genes (Supplementary Figure S4). These elements include transcription factor-binding sites, enhancers and CTCF-binding sites where expression and interaction of these lncRNAs with these regulatory elements in the 3-D genomic space could lead to regulation of many other genes.

We identified two lncRNAs that may potentially be acting as hub genes in Module 2 (PCAT19 and AC093278.2). Both overlap or are next to enhancers and CTCF-binding sites, suggesting a trans mechanism (Supplementary Figure S4). Little is known about their function, although they may have potential as therapeutic targets or biomarkers should they be detectable in circulation. PCAT19 has been shown to negatively regulate p53 in lung cancer [20], whilst p53 has been shown to negatively regulate ischemia-induced angiogenesis [21]. While the function of AC093278.2 has not been described, it is in close proximity to an enhancer that is active in the left ventricle.

Among the remaining lncRNAs in Module 2, CARMN is associated with a super enhancer and has been shown to control cardiac specification and differentiation [22] and AC005550.2 is antisense and overlapping to Homeobox protein MOX-2 MEOX2 (also in Module 2 which regulates cardiac energy metabolism via fatty acid uptake in heart capillary endothelium [23]. Certain protein-coding genes within the module have already been associated with cardiac angiogenesis and vasculogenesis, such as Fms-Related Receptor Tyrosine Kinase 1 (FLT1) [24]. A recent paper [25] proposed that coronary arterial development is regulated by a Dll4-Jag1-EphrinB2 signaling cascade. All three are amongst the genes most strongly associated with ischemia in Module 2.

When lncRNAs have been functionally validated in vivo, they can have a plethora of functions including as guides, with lncRNAs guiding chromatin-modifying enzymes or transcription factors to specific loci; as decoys, binding to transcription factors or miRNAs, preventing them from interacting with their target; as scaffolds, to temporarily assemble protein complexes and cofactors to induce their function; as modulators of 3D organisation of DNA to regulate cell specific gene expression programs; as regulators of formation of nuclear paraspeckles, protein-rich organelles that sequester proteins and RNA to regulate gene expression; as regulators of cellular localization of RNA-binding proteins; and finally by binding the mRNA and either stabilizing them to promote translation or promoting their decay thereby inhibiting translation [26]. From our data, several lncRNAs overlapped cis-eQTLs, including two annotated lncRNAs from Module 1 that highly correlated with their associated gene AC005523.2- FEM1A (discussed previously) and AC011476.3–RDH13. RDH13 encodes a mitochondrial short-chain dehydrogenase/reductase which is suggested to protect mitochondria against oxidative stress [27]. We also found that one of the novel lncRNAs identified RWDD3-DT, overlapped five cis-eQTLs associated with RWDD3, an enhancer of Small Ubiquitin-like Modifier (SUMO) conjugation which modifies proteins post-translation and is involved in heart specific development, metabolism, contractility, and protein quality control [28]. The expression of RWDD3 and RWDD3-DT was correlated (Supplementary Figure S7) and both were present in the WGCNA Module 2. It is possible that eQTL eSNP(s) may affect its secondary structure and ability to bind either to chromatin, a transcription factor, a promoter or the mRNA itself.

Identification of the subcellular localization of an lncRNA (nucleus versus cytoplasm) can give insight into the biological function of the lncRNA. The RNAScope results indicated that all three lncRNAs analyzed (VASH1-AS1, and the novel lncRNA VTCN1-AS (Module 1) and PCAT19 (Module 2)) were located in both the nucleus and the cytoplasm. It is possible that these lncRNAs act in the cytoplasm and the transcripts identified in the nucleus are in the process of being transported to the cytoplasm. The handful of studies available for PCAT19 propose it may function through interactions with microRNAs; however, this still does not rule out either location as miRNAs and Argonaute are also found in both [20,29,30,31]. Additional work is needed to gain insight into the functional role of these lncRNAs.

Our study has several limitations. First, as ischemia was induced by cold cardioplegia and the median cold ischemic time was 74 min, the gene expression changes seen here reflect the immediate response to a relatively short, mild ischemia and lncRNA changes associated with prolonged ischemia or a more severe response (e.g., after myocardial infarction) are not assessed. Second, it is possible some of the changes in gene expression may also be due to a cardioplegic cold response, rather than ischemia per se. Third, although the samples were paired, providing improved statistical power compared with independent samples, a larger sample size may have enabled identification of a larger number of novel lncRNA transcripts and lncRNAs associated with ischemia. Fourth, the function of most lncRNAs is unknown and the mechanisms by which the lncRNAs identified here may influence ischemia requires further analysis.

## 4. Materials and Methods

The Partners HealthCare Institutional Review Board approved this study and written informed consent was obtained from each patient [7]. A total of 85 patients undergoing aortic valve replacement surgery with cardiopulmonary bypass were prospectively enrolled as previously described [7]. Punch biopsies were collected from the left ventricular (LV) apex pre-ischemia (immediately upon initiation of cardiopulmonary bypass) at which time the heart was arrested with cold blood cardioplegia, and post-ischemia (after a median of 74 min). Total RNA was isolated (yielding ≈ 3–5 μg) and ribosomal RNA was removed with poly-T oligo beads as previously described [7]. Paired-end sequencing (2 × 100 bp) was performed on the Illumina HiSeq 2000 (Illumina, San Diego, CA, USA) [7].

### 4.1. Illumina Sequencing and QC Analysis

The pipeline developed in this study is freely available to download from Github https://github.com/zoeward-nz/PhD (accessed on September 2021). The raw reads in FASTQ format were filtered to remove adapter sequences and low-quality bases using Trimmomatic v0.36 [32]. A sliding window was used to trim bases with a Phred quality score <20 with a minimum length filter of 36 bases. Quality trimmed raw reads were aligned to the human reference genome sequence (GRCh38.p12) using STAR2 v2.6.0 [33] with ENCODE parameters (https://github.com/alexdobin/STAR/blob/master/doc/STARmanual.pdf accessed on September 2019) using GENCODE v29 [34] as a reference annotation. The aligned reads were assembled into transcripts using Stringtie v1.3.3 [35] to produce GTF files for each sample. Transcripts with a minimum of 0.1 Fragments Per Kilobase of transcript per Million reads (FPKM) from individual sample GTF files were merged together to form a single set of nonredundant transcripts using Stringtie merge. This merged GTF was then compared against the reference genome using gffcompare v0.10 (https://ccb.jhu.edu/software/stringtie/gffcompare.shtml accessed on September 2019) to classify annotated and novel transcripts.

Gffread (http://ccb.jhu.edu/software/stringtie/gff.shtml accessed on September 2019) was used to generate a FASTA file from the merged Stringtie GTF which was used with Salmon v0.10 [36] to generate quantification estimates transcripts. Principal Component Analysis (PCA), MultiQC metrics [37] and the R package TissueEnrich [38] were used to identify samples with expression patterns inconsistent with the human left ventricle. For three samples, one of the pairs had expression profiles from a mixture of tissues and one sample failed MultiQC metrics (Supplementary Figures S1–S3) and these were excluded. This left a total of 81 paired samples for differential expression analysis.

### 4.2. Nanopore Long-Read Sequencing

To confirm the authenticity of novel lncRNAs detected by Illumina sequencing, an independent sequencing platform, using the gridION Oxford Nanopore Technologies (Nanopore), was used to sequence left ventricle tissue pooled from 8 heart donors from the Cleveland Clinic Tissue Bank.

Heart tissue from the left ventricular free wall of organ donors (*n* = 108) was collected by the Human Tissue Core Facility at the Kaufman Centre for Heart Failure, Cleveland Clinic, between August 1993 and May 2005. The Human Tissue Core Facility holds explanted human hearts from heart transplant recipients and healthy heart tissue from unmatched organ donors (Cleveland Clinic IRB ethics approval: IRB 2378). Tissue was provided to the Christchurch Heart Institute in collaboration with Professor Christine Moravec and Ms Wendy Sweet (New Zealand Health and Disability Ethics Committee approval CTR/03/11/199/AM03). A single cDNA library was prepared with the cDNA-PCR Sequencing Nanopore SQK-PCS109 kit according to the manufacturer’s instructions (PCS_9085_v109_revB_04Feb2019). Briefly, total RNA extracted from left ventricle tissue from 8 donors was pooled (eRIN ≥ 8, Agilent Tapestation) and 50 ng combined with 0.05 ng (1%) RNA sequins (Mix A) as internal controls. The recommended 14 PCR cycles was used to amplify full-length poly-A RNA transcripts prior to cDNA synthesis.

Prepared libraries were run on R9.4 flowcells using the GridION platform, reads were base-called using Guppy base-calling software (v3.03 High accuracy). Full-length transcripts were identified using (default settings) Pychopper (https://github.com/nanoporetech/pychopper accessed on September 2019) and mapped to a combined index comprising the human genome (hg38) and the synthetic RNA standards sequins in silico chromosome (chrIS) [39] using Minimap2 (v2.17–r941, parameters: –ax splice –secondary = no.) [40]. Primary alignments were selected with Samtools (http://www.htslib.org/doc/samtools.html parameters: view –F 2304 accessed on September 2019) and processed using the Pinfish suite (https://github.com/nanoporetech/pinfish, parameters: −*p* 1.0 − c 3 − d 10 − e 30).

### 4.3. Identification and Validation of Novel lncRNAs

An in-house pipeline was developed for the detection of putative novel lncRNA transcripts from Illumina and Nanopore sequencing using the following inclusion criteria: (i) for the Illumina short-read technology to eliminate very low level transcripts, a filter of read counts > 0 in at least 50% of the samples was implemented; (ii) length > 200 nucleotides; (iii) multi-exonic; (iv) were either gffcompare class codes ‘u’, ‘i’, ‘x’, ‘y’ with respect to reference annotation GENCODE v.29; (v) lacked coding potential (as assessed by the Coding Potential Assessment Tool (CPAT) v1.2.3 using recommended defaults [41] (Figure 2) aligned to the 22 autosomes or X chromosome. Novel transcripts from both technologies were compared against each other using gffcompare and transcripts that had a complete match of intron chains (class code ‘=’) were deemed to be validated novel lncRNAs.

### 4.4. Conservation of Novel lncRNAs

Evolutionary conservation of novel lncRNAs was assessed using precomputed nucleotide level calculations of evolutionary selection from the phastCons score. This score identifies evolutionary conserved elements in multiple-aligned sequences and assigns each base a score between 0 and 1 (higher scores indicate greater conservation) [42]. A bigwig file containing phastCons base-by-base conservation scores across 20 mammalian species was downloaded from the UCSC site (http://hgdownload.soe.ucsc.edu/goldenPath/hg38/phastCons20way/hg38.phastCons20way.bw accessed on September 2019) and converted to bed format using the bigWigToBedGraph tool (http://hgdownload.soe.ucsc.edu/admin/exe/ accessed on September 2019). Novel lncRNAs, annotated lncRNAs and coding mRNAs were aligned to the phastCons bed file and a mean phastCons score was extracted for each exon using bedtools map (bedtools map -a genes.bed -b phastCons.bedgraph -c 4 -o mean). To compare the conservation profile of exon sequences between novel lncRNAs, annotated lncRNAs and mRNAs, the frequency of scores were plotted for each group separately.

### 4.5. Differential Expression

To identify differentially expressed novel lncRNAs, tximport v1.9.12 [43] was used to import gene and transcript level count matrices into DESEQ2 v1.24.0 [44]. Pre- to post-ischemia paired samples were compared at the gene level for annotated mRNAs and lncRNAs, and at the transcript level for putative novel lncRNAs. Conservative selection criteria was used to identify a ‘high-confidence’ set of differentially expressed genes/transcripts for downstream analysis: (i) adjusted *p*-value < 0.001 after adjustment for multiple comparisons (Benjamini–Hochberg), (ii) absolute fold change >1.2 (post-ischemic/pre-ischemic) and (iii) for annotated genes, at least 90% of each pre- and post- group had to have an expression level of at least 0.5 transcripts per million (TPM).

### 4.6. WGCNA: Weighted Correlation Network Analysis

To identify clusters (modules) of highly correlated genes (including novel lncRNAs) that share a similar pattern of expression across patients and may be co-regulated, an unsigned weighted gene co-expression network analysis was performed using the WGCNA package in R [45]. WGCNA assigns genes to clusters based on correlation and shared network neighbours. Each module is represented by a weighted average of the expression level of all genes within the module, referred to as the module eigengene (kME). Each module eigengene was then tested to identify modules with the strongest association to ischemia. Each gene is assigned a module membership which is defined as the correlation of the module eigengene and the gene expression profile. The module membership reflects the gene’s intramodular interconnectivity. In this way we can identify highly interconnected ‘hub’ genes that may be driving the response to ischemia. Each module was filtered to only include DESEQ2 differentially expressed genes. The novel lncRNA and the putative hub lncRNAs were viewed in Ensembl Genome Browser (https://www.ensembl.org accessed on September 2019) with the regulatory track turned on. This shows enhancers, transcription factor-binding sites, CCCTC-binding factor (CTCF)-binding sites (thought to regulate the 3D structure of chromatin), promoters and promoter flanks.

### 4.7. Ingenuity Pathway Analysis (IPA)

To identify the molecular pathways, networks and related biological functions, differentially expressed genes within those modules identified as significantly associated with ischemia, were assessed using the Core Analysis Workflow with Ingenuity Pathway Analysis (IPA) software (http://www.ingenuity.com accessed on September 2019, Qiagen, Redwood City, CA, USA). IPA gives a z-score which represents a statistical measure of the match between the expected and observed direction of gene expression. Any z-score > 2 (indicating pathway activation) or < −2 (indicating pathway repression) was considered to be potentially biologically meaningful.

### 4.8. Expression Quantitative Trait Locus (eQTLs)

Expression quantitative trait locus (eQTLs) analysis investigates associations between SNPs and gene expression levels. Overlap of both novel and annotated lncRNA exon coordinates with cis-eQTL SNPs from GTEX v.8 left ventricle (https://gtexportal.org/home/datasets accessed on September 2019) was assessed using Bedtools Intersect (https://bedtools.readthedocs.io/en/latest/content/tools/intersect.html accessed on September 2019). LncRNAs found to be associated with an eQTL SNP were analyzed to see whether the lncRNA and eQTL gene were members of the same WGCNA module (and therefore potentially co-regulated).

### 4.9. Subcellular Localization of lncRNAs

The subcellular localization of two annotated lncRNAs and a novel lncRNA associated with the ischemia-associated modules from WGCNA was investigated using in situ hybridization with RNAscope (Advanced Cll Diagnostics (ACD), Newark, CA, USA). Briefly, this technology uses a specific double ‘Z’ shaped probe to hybridize to the target RNA sequence (approximately 18–25 bases). The probe is then bound by amplifier probes, which bind the chromogenic label (fast-red + alkaline phosphatase). We used custom RNAscope probes for the two annotated lncRNAs (PCAT 19 and VASH1-AS) and one novel lncRNA (VTCN1-AS). A negative control dapB probe was used as well as a positive control NEAT1, a lncRNA demonstrated to be expressed predominantly in the nucleus.

Formalin-Fixed Paraffin-Embedded (FFPE) left ventricle tissue glass slides were baked for 1 h at 65 °C. In situ hybridization analysis was carried out using the RNAscope 2.5 HD Detection Reagent-RED kit (ACDBio). All analyses were carried out according to the manufacturer’s instructions using PCAT 19, VASH1-AS and VTCN1-AS probes. Slides were analyzed and Tagged Image Format (.TIF) images captured using a Zeiss Apotome Microscope and AxioVersion v4.5 and Apotome software.

## 5. Conclusions

This study has established a bioinformatics pipeline and methodology for identifying and validating putative novel lncRNAs in human heart tissue. We have identified ten novel lncRNAs in the human left ventricle, one of which may play a role in the early response to ischemia. Our data suggest that lncRNAs are among the first genes to respond to ischemic changes in the left ventricle. Several lncRNAs appear to act as hub genes, potentially coordinating the expression of multiple genes. These and other differentially expressed lncRNAs may represent novel therapeutic targets or candidate biomarkers for early myocardial dysfunction.

## Figures and Tables

**Table 1:** Information on the 10 novel lncRNAs detected with our pipeline.

Putative Name	Chr’me	Start	Stop	Strand	# Exons	Mean TPM
VTCN1-AS	chr1	117128696	117143589	+	2	1.68
LINC02934	chr2	37489457	37605898	+	3	0.22
LINC02935	chr3	15894181	16137554	+	4	0.32
ADCY5-AS	chr3	123335278	123338361	+	3	0.47
LINC02936	chr5	107778856	107781422	+	2	0.34
LINC02937	chr6	157328269	157363141	+	4	0.37
LINC02938	chr8	94223489	94228144	−	3	0.30
PDGFD-AS	chr11	104071819	104093201	+	4	0.33
DHRS1-AS	chr14	24271210	24299055	+	3	2.03
RWDD3-DT	chr1	95247358	95256066	+	2	0.36

## Data Availability

Data will be made available upon request.

## Supplementary Materials

The following are available online at https://www.mdpi.com/article/10.3390/ijms222111324/s1.
