# Supplementary material for: Novel and Annotated Long Noncoding RNAs Associated with Ischemia in the Human Heart
Source: Int J Mol Sci. 2021 Oct 20;22(21):11324. doi: 10.3390/ijms222111324 (PMC8583240; doi:10.3390/ijms222111324)
Supplement: Supplementary file 1 [file ijms-22-11324-s001.zip › ijms-1417197-supplementary.pdf]

Supplementary Materials

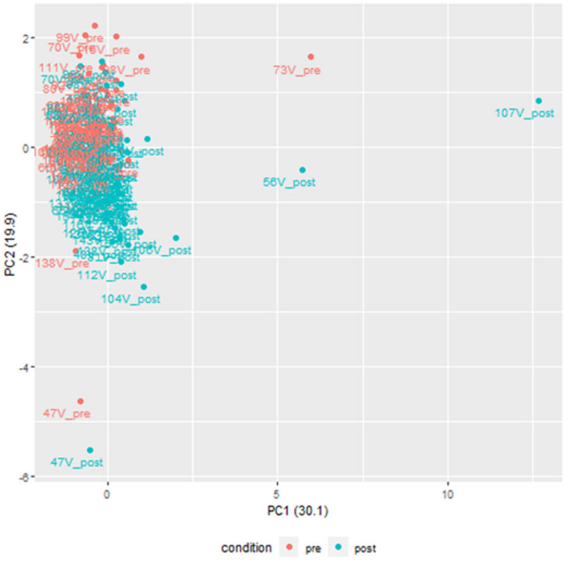

Supplementary Figure S1: Principal Component Analysis (PCA) on the transcriptome of the samples. Red indicates pre-ischemic samples; blue indicates post-ischemic samples.

Supplementary Figure S2. MultiQC plot showing the aberrant fragment length distribution generated during library preparation of sample ID 47 pre- and post-ischemia. This sample and the samples from Supplementary Figure 3 were dropped from the analysis.

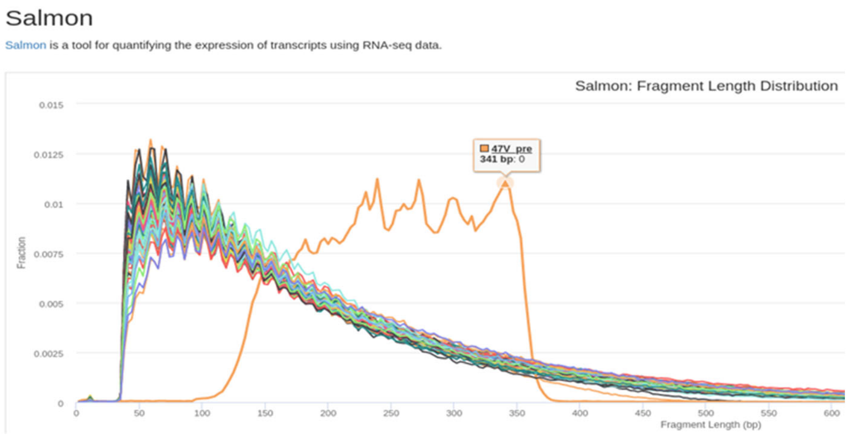

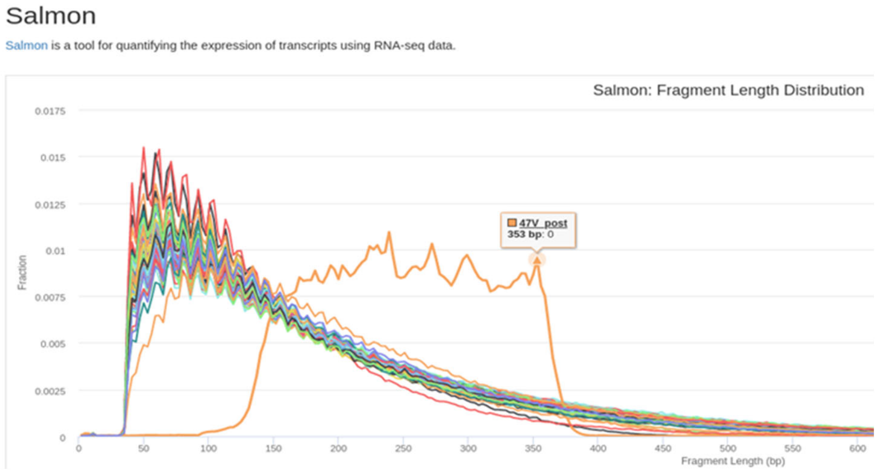

Supplementary Figure S3. R package TissueEnrich plots showing the expression of tissue expression genes. Normal plots (56V_pre, 73V_post and 107_pre) show the highest gene expression from heart muscle followed by a lower expression from skeletal muscle. Samples 56V_post, 73V_pre and 107V_post show aberrant gene expression with very low gene expression from heart tissue with expression from other tissue. Note the y-axes are different for the aberrant samples.

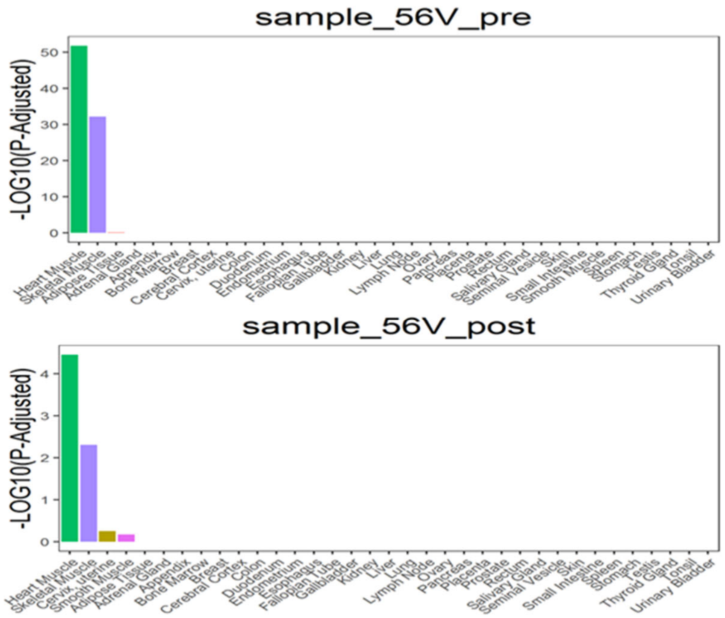

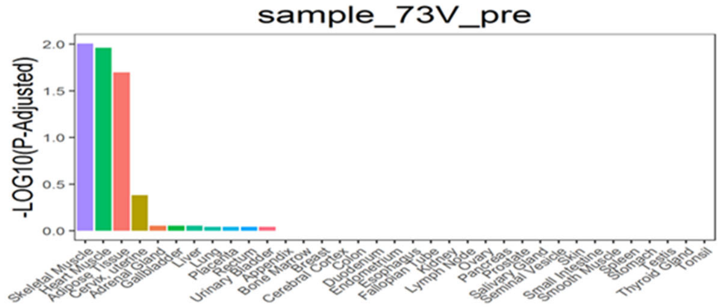

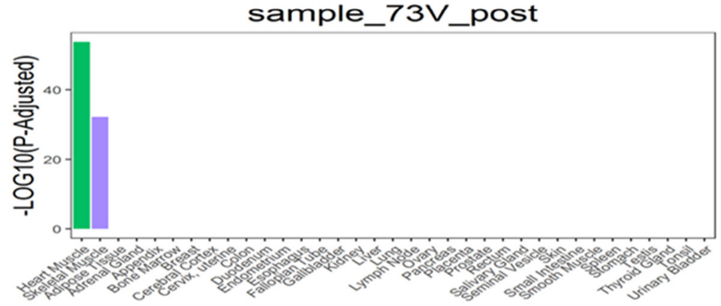

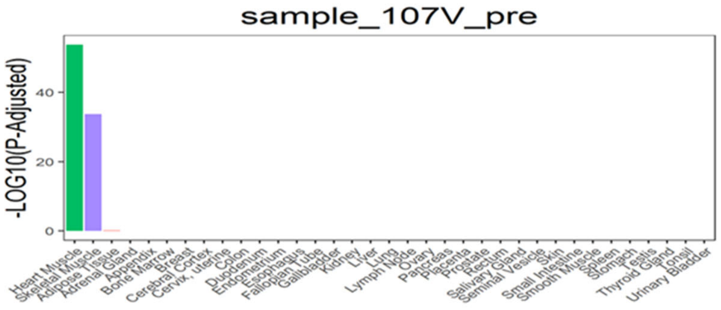

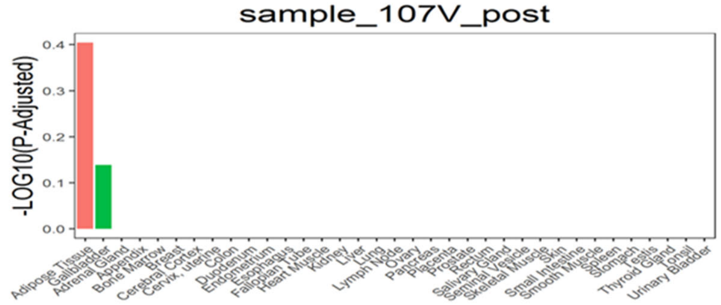

Supplementary Figure S4. Screen shots from Ensembl Genome Broswer showing overlap of regulatory sites. A–D) The novel lncRNA identified in this study and the three putative hub lncRNAs from module 1; E–F) the two putative hub lncRNAs from module 2.

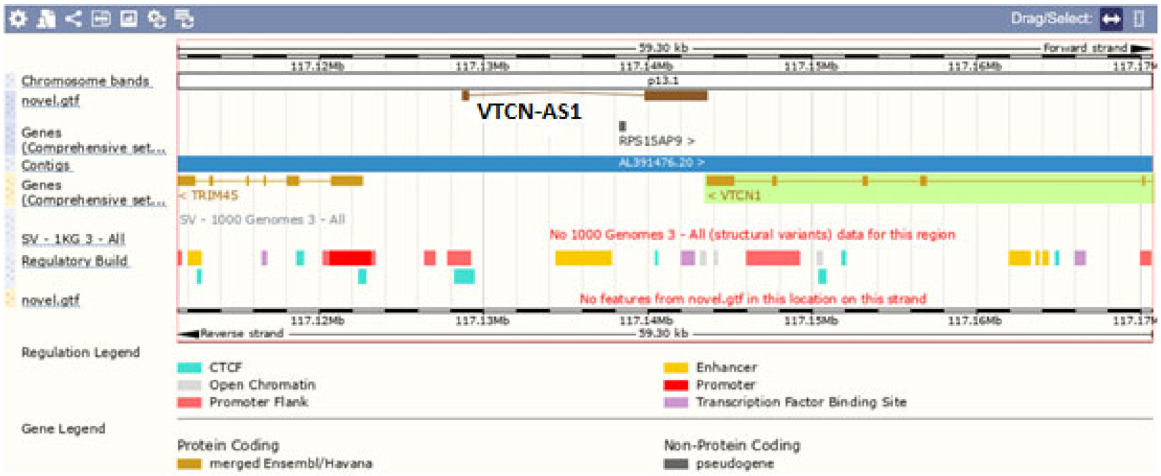
Drag/Select:
59.30 kb
Forward strand
117.12Mb
117.13Mb
117.14Mb
117.15Mb
117.16Mb
p13.1
Chromosome bands
novel.gtf
VTCN-AS1
Genes (Comprehensive set...
RPS15AP9 >
Contigs
AL391476.20 >
Genes (Comprehensive set...
< TRIM45
< VTCN1
SV - 1000 Genomes 3 - All
SV - 1KG 3 - All
No 1000 Genomes 3 - All (structural variants) data for this region
Regulatory Build
novel.gtf
No features from novel.gtf in this location on this strand
Reverse strand
Regulation Legend
CTCF
Open Chromatin
Promoter Flank
Enhancer
Promoter
Transcription Factor Binding Site
Gene Legend
Protein Coding
merged Ensembl/Havana
Non-Protein Coding
pseudogene

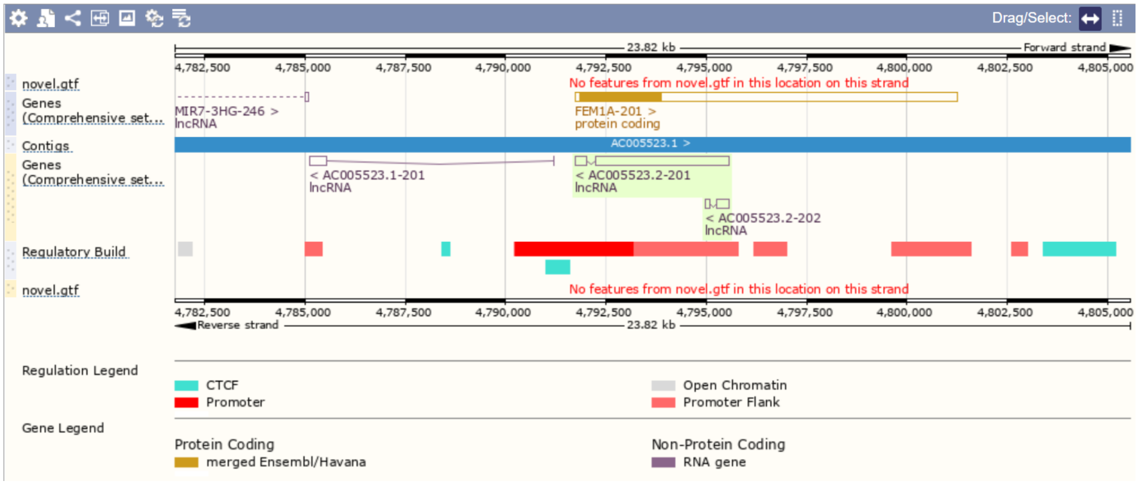
Drag/Select:
23.82 kb
Forward strand
4,782,500
4,785,000
4,787,500
4,790,000
4,792,500
4,795,000
4,797,500
4,800,000
4,802,500
4,805,000
novel.gtf
No features from novel.gtf in this location on this strand
Genes (Comprehensive set...
MIR7-3HG-246 >
lncRNA
FEM1A-201 >
protein coding
Contigs
AC005523.1 >
Genes (Comprehensive set...
< AC005523.1-201
lncRNA
< AC005523.2-201
lncRNA
< AC005523.2-202
lncRNA
Regulatory Build
novel.gtf
No features from novel.gtf in this location on this strand
Reverse strand
Regulation Legend
CTCF
Promoter
Open Chromatin
Promoter Flank
Gene Legend
Protein Coding
merged Ensembl/Havana
Non-Protein Coding
RNA gene

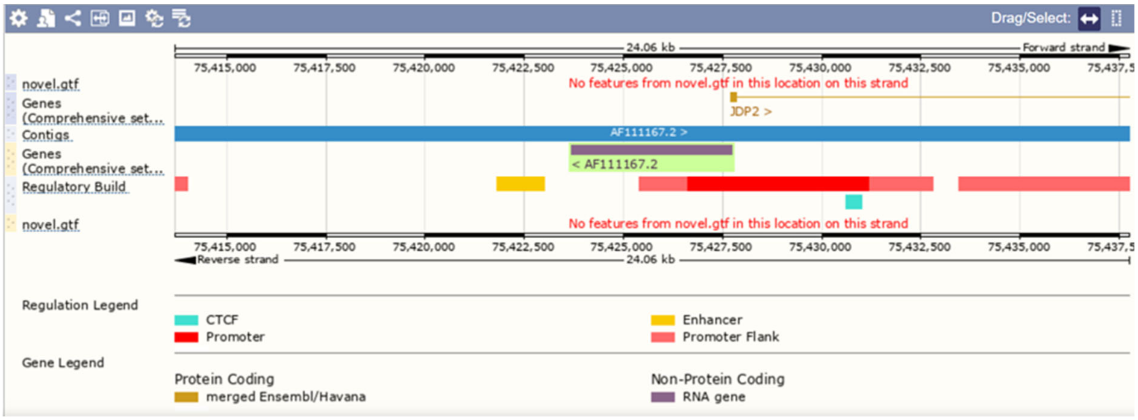
Drag/Select:
24.06 kb
Forward strand
75,415,000
75,417,500
75,420,000
75,422,500
75,425,000
75,427,500
75,430,000
75,432,500
75,435,000
novel.gtf
No features from novel.gtf in this location on this strand
Genes (Comprehensive set...
JDP2 >
Contigs
AF111167.2 >
Genes (Comprehensive set...
< AF111167.2
Regulatory Build
novel.gtf
No features from novel.gtf in this location on this strand
Reverse strand
Regulation Legend
CTCF
Promoter
Enhancer
Promoter Flank
Gene Legend
Protein Coding
merged Ensembl/Havana
Non-Protein Coding
RNA gene

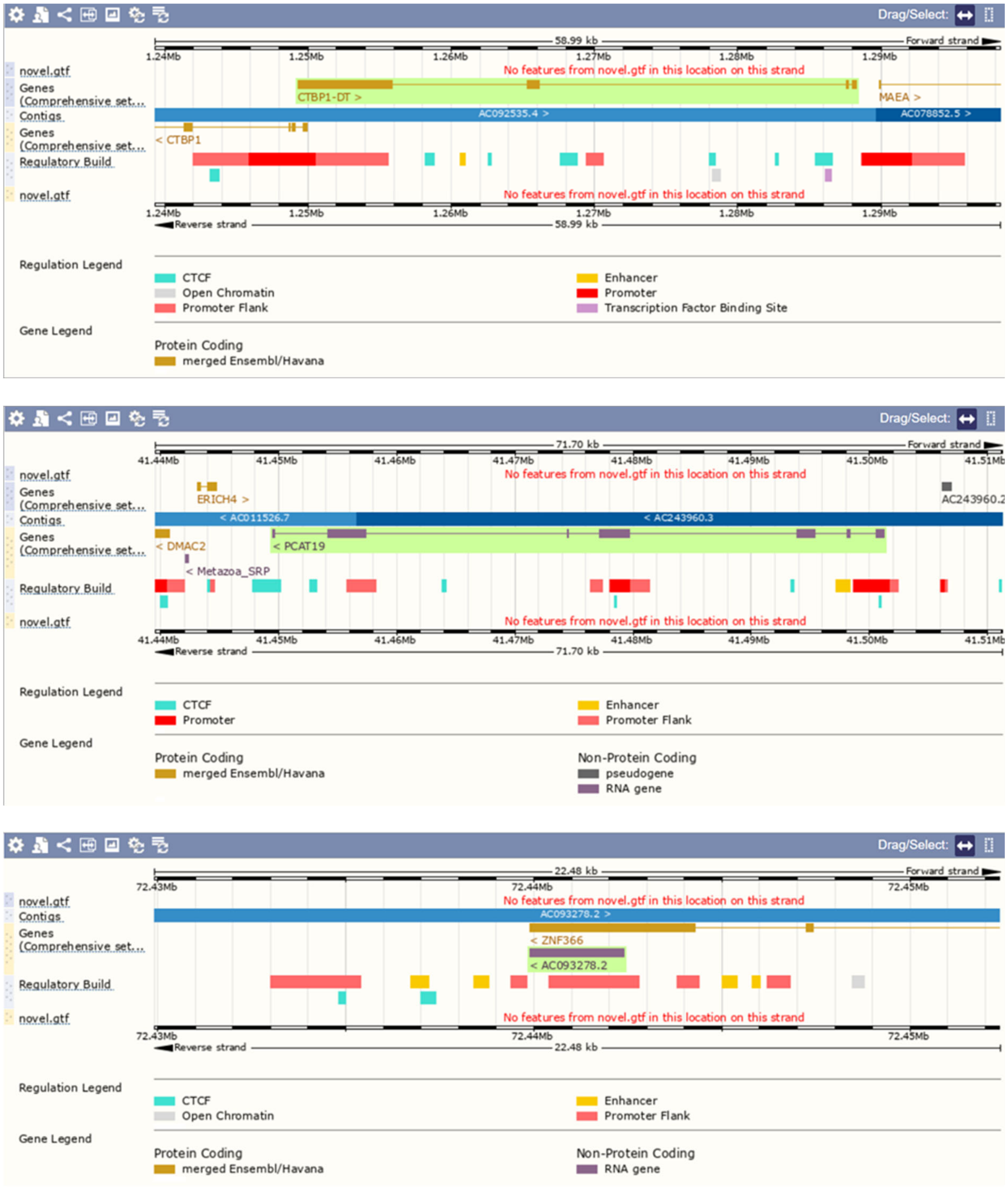

Supplementary Figure S5. Sequin controls. We carried out isoform-level quantification on the internal sequin controls using Salmon to assess the limit of detection. We found a strong correlation between measured abundance (transcripts per million TPM) and input concentration (R2 = 0.82, slope = 1.25) and a limit of detection of 0.0194 attomoles/μL.

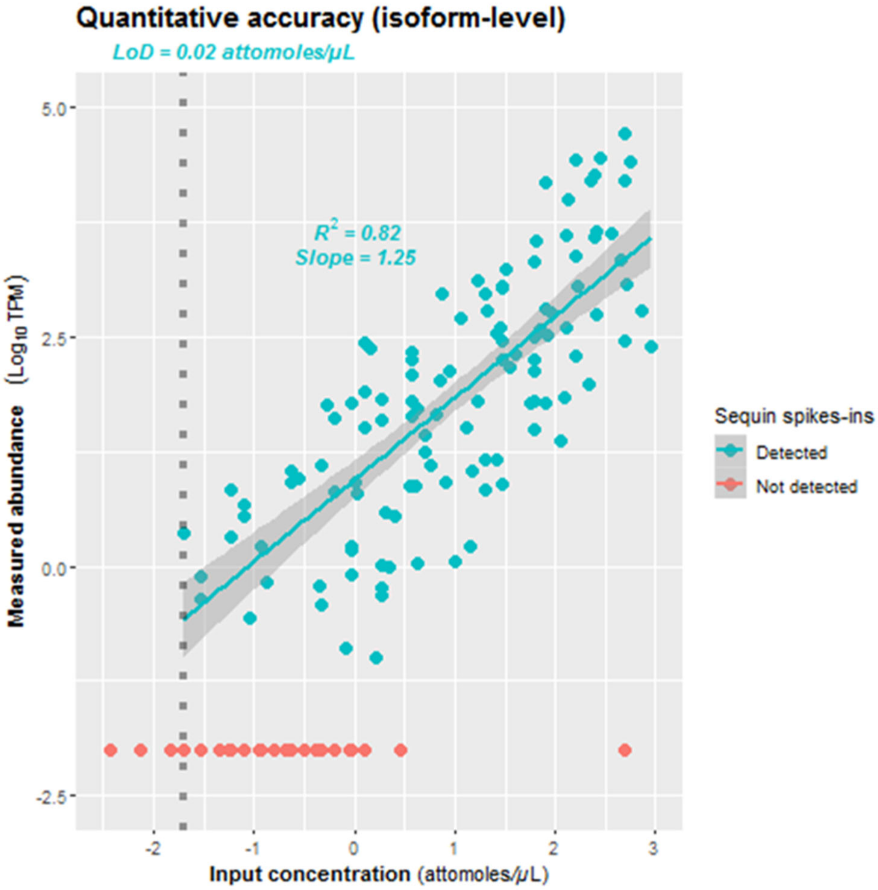

Supplementary Figure S6. Module-trait relationships predicted by Weighted Gene-Correlated Network Analysis (WGCNA). Each cell shows the correlation and p-value between the module eigengene (row) and the trait (column). The table is color-coded by correlation according to the color legend.

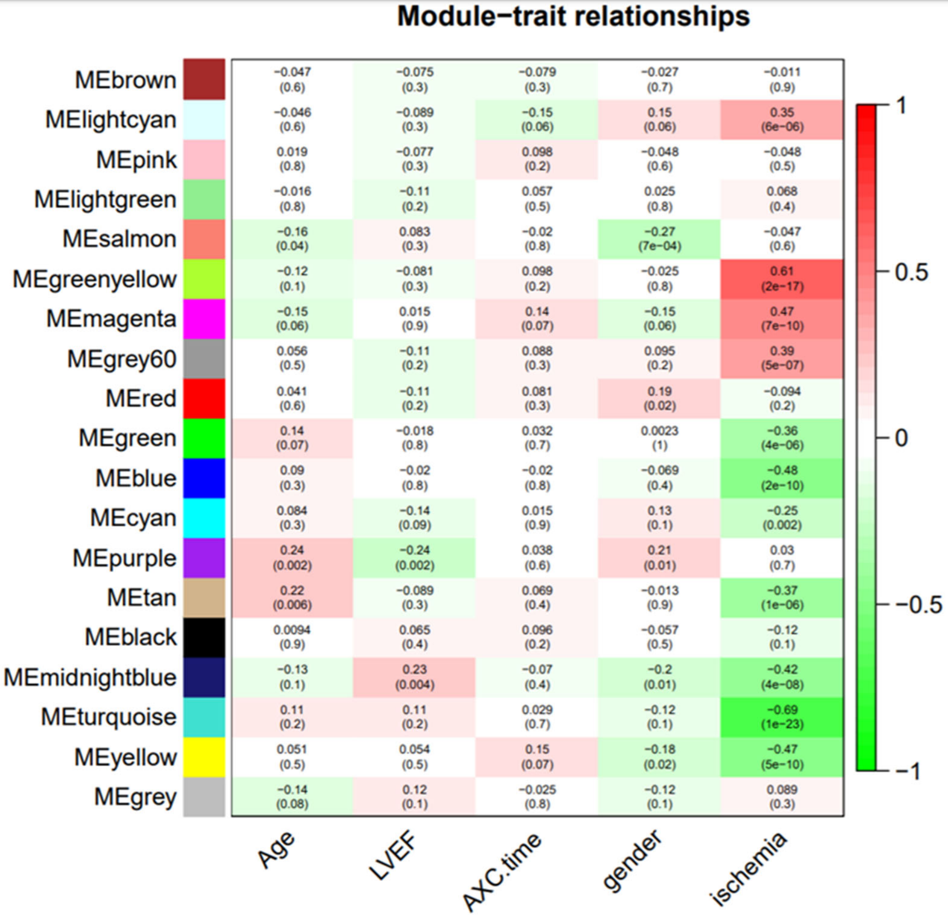

Supplementary Figure S7. Pearson correlation of lncRNA-mRNA pairs AC005523.2- FEM1A (left) and AC011476.3—RDH13 (right) where the lncRNA also overlapped cis-eQTLs associated with the mRNA.

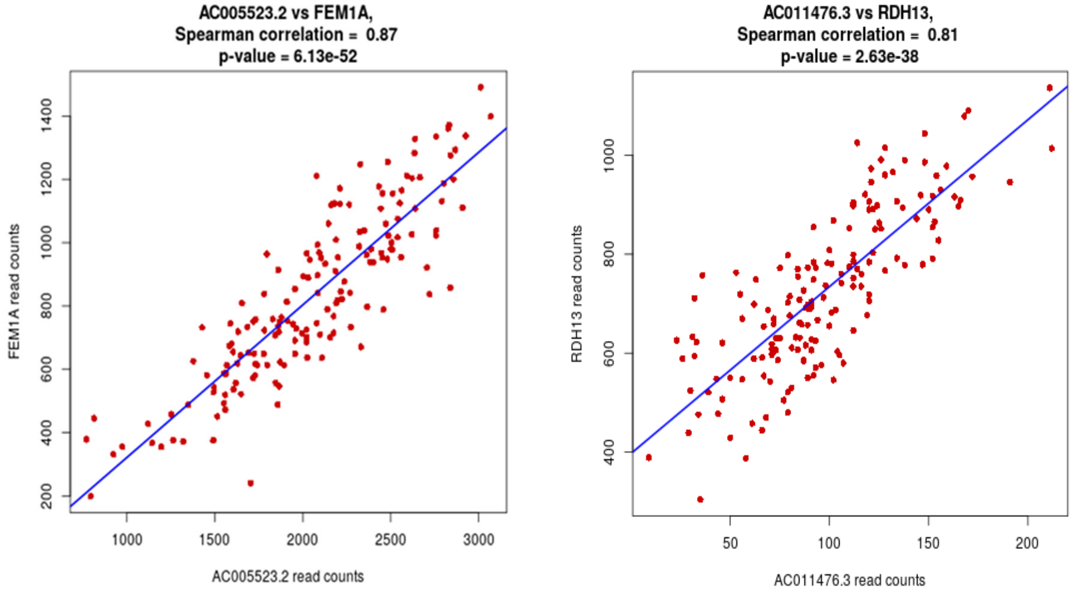

Supplementary Figure S8. Pearson correlation of novel lncRNA-mRNA pair RWDD3-DT—RWDD3. RWDD3-DT overlapped cis-eQTLs associated with RWD Domain-Containing Sumoylation Enhancer (RWDD).

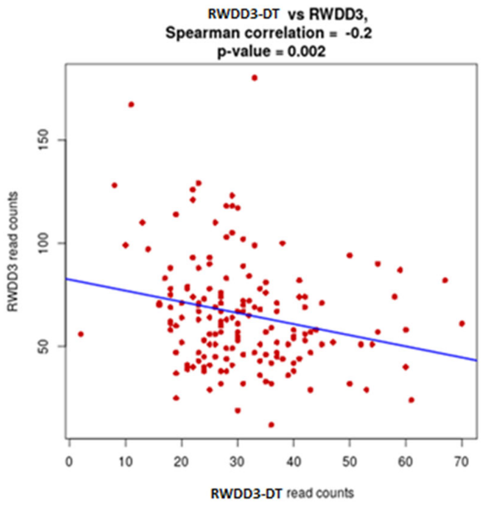

Supplementary Table S1 The exon co-ordinates for the 10 novel lncRNAs identified by the pipeline.

Putative Name	Feature	Chromosome	Start	Stop	Strand
VTCN1-AS	transcript	chr1	117128696	117143589	+
	exon	chr1	117128696	117129041	+
	exon	chr1	117139815	117143589	+
LINC02934	transcript	chr2	37489457	37605898	+
	exon	chr2	37489457	37489581	+
	exon	chr2	37536995	37537151	+
	exon	chr2	37605236	37605898	+
LINC02935	transcript	chr3	15894181	16137554	+
	exon	chr3	15894181	15894285	+
	exon	chr3	15946784	15946855	+
	exon	chr3	15947135	15947192	+
	exon	chr3	16137391	16137554	+
ADCY5-AS	transcript	chr3	123335278	123338361	+
	exon	chr3	123335278	123335579	+
	exon	chr3	123336338	123336400	+
	exon	chr3	123337544	123338361	+
LINC02936	transcript	chr5	107778856	107781422	+
	exon	chr5	107778856	107779025	+
	exon	chr5	107780752	107781422	+
LINC02937	transcript	chr6	157328269	157363141	+
	exon	chr6	157328269	157328448	+
	exon	chr6	157332280	157332451	+
	exon	chr6	157361737	157361784	+
	exon	chr6	157363066	157363141	+
LINC02938	transcript	chr8	94223489	94228144	-
	exon	chr8	94223489	94225595	-
	exon	chr8	94226722	94226806	-
	exon	chr8	94228039	94228144	-
PDGFD-AS	transcript	chr11	104071819	104093201	+
	exon	chr11	104071819	104072232	+
	exon	chr11	104084012	104084205	+

	exon	chr11	104085349	104085425	+
	exon	chr11	104092371	104093201	+
DHRS1-AS	transcript	chr14	24271210	24299055	+
	exon	chr14	24271210	24271310	+
	exon	chr14	24298614	24298873	+
	exon	chr14	24299027	24299055	+
RWDD3-DT	transcript	chr1	95247358	95256066	+
	exon	chr1	95247358	95247934	+
	exon	chr1	95252486	95256066	+

Supplementary Table S2. The top five disease or functions predicted by Ingenuity Pathway Analysis (IPA) (sorted by z-score) associated with the two modules most associated with ischemia (WGCNA).

Categories	Module	Diseases or Functions Annotation	*p*-value	Predicted Activation State	Activation z-Score
**Cell Death and Survival**	**1**	Necrosis	$6.47 * 10^{-9}$	Increased	3.4
**Organismal Survival**	**1**	Morbidity or mortality	$9.2 * 10^{-8}$	Increased	12.7
**Cell Death and Survival**	**1**	Apoptosis	$9.03 * 10^{-8}$	Increased	4.1
**Organismal Survival**	**1**	Organismal death	$5.58 * 10^{-8}$	Increased	12.7
**Organismal Injury and Abnormalities**	**1**	Organ Degeneration	$4.3 * 10^{-6}$	Increased	4.3
**Cardiovascular System Development and Function**	**2**	Angiogenesis	$6.31 * 10^{-13}$	Increased	4.4
**Cardiovascular System Development and Function**	**2**	Development of vasculature	$1.02 * 10^{-13}$	Increased	4.4
**Cardiovascular System Development and Function**	**2**	Vasculogenesis	$1.23 * 10^{-12}$	Increased	4.3
**Hematological System Development and Function**	**2**	Hematopoiesis of mononuclear leukocytes	$9.75 * 10^{-7}$	Increased	3.4
**Hematological System Development and Function**	**2**	Quantity of lymphocytes	$1.42 * 10–^{6}$	Increased	3.7
